# Semiconducting polymer nano-radiopharmaceutical for combined radio-photothermal therapy of pancreatic tumor

**DOI:** 10.1186/s12951-021-01083-0

**Published:** 2021-10-24

**Authors:** Xiumin Shi, Qing Li, Chuan Zhang, Hailong Pei, Guanglin Wang, Hui Zhou, Longfei Fan, Kai Yang, Bo Jiang, Feng Wang, Ran Zhu

**Affiliations:** 1 State Key Laboratory of Radiation Medicine and Protection, School of Radiation Medicine and Protection & School for Radiological and Interdisciplinary Sciences (RAD-X), Collaborative Innovation Center of Radiation Medicine of Jiangsu Higher Education Institutions, Soochow University, Suzhou, 215123 Jiangsu China; 2 Department of Nuclear Medicine, Nanjing First Hospital, Nanjing Medical University, Nanjing, 210006 China; 3 Department of Neuro-Oncology, Cancer Center, Beijing Tiantan Hospital, Capital Medical University, Beijing, 100071 China

**Keywords:** Semiconducting polymer nanoparticles (SPNs), Radiopharmaceuticals, Pancreatic cancer, Combined therapy, Radiotherapy, Photothermal therapy

## Abstract

**Background:**

Pancreatic ductal adenocarcinoma (PDAC) is a devastatingly malignant tumor with a high mortality. However, current strategies to treat PDAC generally have low efficacy and high side-effects, therefore, effective treatment against PDAC remains an urgent need.

**Results:**

We report a semiconducting polymer nano-radiopharmaceutical with intrinsic photothermal capability and labeling with therapeutic radioisotope ^177^Lu (^177^Lu-SPN-GIP) for combined radio- and photothermal therapy of pancreatic tumor. ^177^Lu-SPN-GIP endowed good stability at physiological conditions, high cell uptake, and long retention time in tumor site. By virtue of combined radiotherapy (RT) and photothermal therapy (PTT), ^177^Lu-SPN-GIP exhibited enhanced therapeutic capability to kill cancer cells and xenograft tumor in living mice compared with RT or PTT alone. More importantly, ^177^Lu-SPN-GIP could suppress the growth of the tumor stem cells and reverse epithelial mesenchymal transition (EMT), which may greatly reduce the occurrence of metastasis.

**Conclusion:**

Such strategy we developed could improve therapeutic outcomes over traditional RT as it is able to ablate tumor with relatively lower doses of radiopharmaceuticals to reduce its side effects.

**Graphical abstract:**

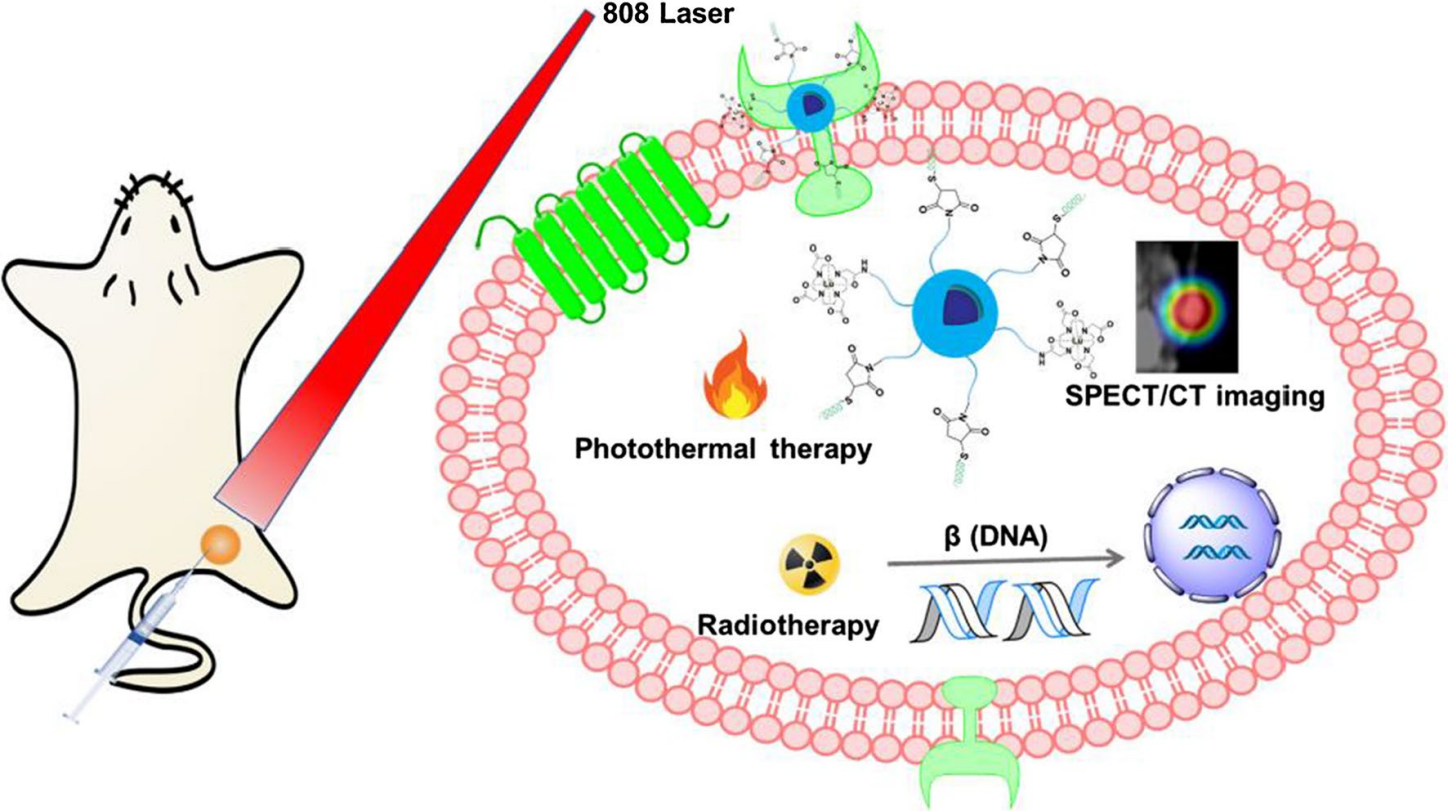

**Supplementary Information:**

The online version contains supplementary material available at 10.1186/s12951-021-01083-0.

## Introduction

Pancreatic ductal adenocarcinoma (PDAC), as the most common type of pancreatic tumors, has poor prognosis and high mortality with 5-year overall survival rate less than 10% [1]. Such low survival rate is commonly caused by rapid tumor progression, easy metastasis and the absence of early symptom of PDAC [2–4]. Resultantly, the majority of patients with PDAC are diagnosed at advanced or metastatic stage and thus are refractory through surgical resection [5]. At such advanced stage, other therapeutic strategies such as chemotherapy have limited efficacy due to its high chemoresistance [6]. Therefore, the high aggressiveness, metastasis and chemoresistance of PDAC urge researchers to exploit new and more effective therapeutic strategies to improve the survival of PDAC patients.

Combined therapy provides new opportunities for effective cancer treatment by utilizing the complementary advantages of different therapeutic methods [7, 8]. For instance, radiotherapy (RT), one of the most effective treatment strategies, has been extensively used in clinical cancer treatment [9–12]. However, some cancers including pancreatic cancer are relatively resistant to RT due to their inherent hypoxic microenvironment within tumor [13]. To overcome this limitation, the combination of RT with other treatment modalities is considered as a promising method to improve therapeutic efficiency. In recent years, various therapeutic modalities such as photothermal therapy (PTT), photodynamic therapy, and immunotherapy have been developed to combine with RT for improving the tumor cell killing effects [14–16]. Among these therapeutic modalities, PTT, which capitalizes on photothermal effect that converts absorbed near-infrared light energy into heat [17–19], has emerged as a potential treatment for cancers due to its high specificity, minimal wound and low side-effects [20]. It has been demonstrated that appropriate hyperthermia not only has the ability to kill the tumor cells that are less sensitive to RT but also can accelerate intratumoral blood flow, which leads to the improvement of oxygen level in tumor and thus relieves the resistance of tumor cells to RT [21, 22]. Therefore, by virtue of the merits, the combination of PTT with RT endows enhanced therapeutic effect against tumors. To date, a series of nanoagents integrating RT with PTT have been developed but have not been explored for PDAC treatment [23, 24]. Besides, most of these reported nanoagents are based on inorganic materials, leading to potential toxicity and impeding their clinical application [25].

Semiconducting polymer nanoparticles (SPNs) composed of completely organic compounds have been emerged as photothermal agents for tumor therapy [26–29]. Compared with other inorganic nanomaterials such as gold nanorods and carbon nanotubes, SPNs possess unique characteristics such as better biocompatibility, higher NIR absorption and photothermal conversion efficiency, feasibility and controllability [30–34]. In particular, SPNs not only allow for convenient chemical engineering of backbones and side chains for on-demand functionalization, but also are conducive to nanoengineering to achieve size or morphology controllability [35]. To date, the utilization of SPNs for PTT has been fully explored, but combined therapy are rarely reported [10, 36, 37].

**Scheme 1:**
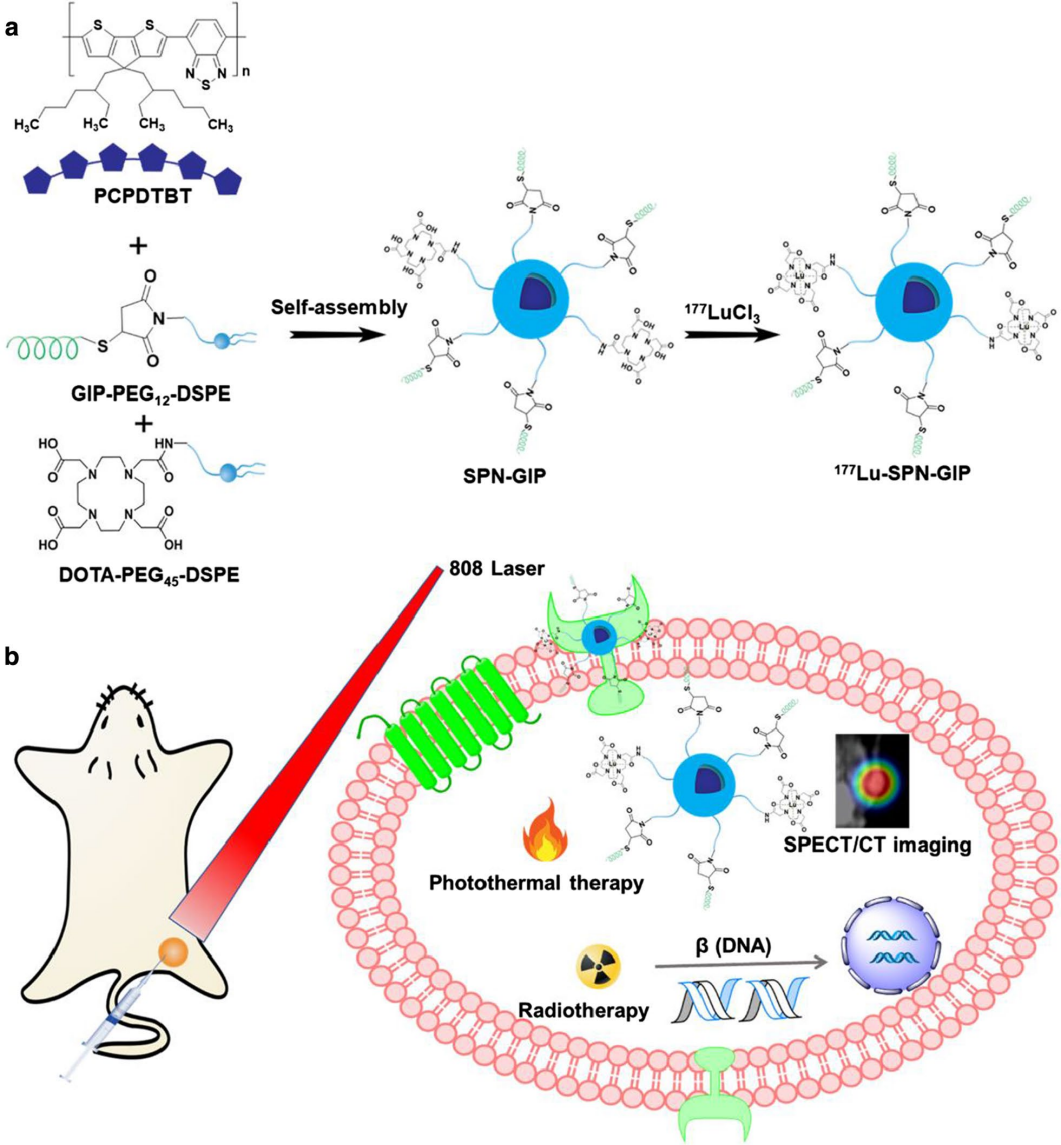
**a** Synthesis and characterization of SPN-GIP and ^177^Lu-SPN-GIP. **b** Representative schematic illustration of the fabrication of SPNs and combined RT and PTT

In this study, we develop a ^177^Lu-labeled semiconducting polymer nano-radiopharmaceutical with glucose-dependent insulinotropic polypeptide (GIP) as tumor targeting group (^177^Lu-SPN-GIP) for combined RT and PTT of PDAC (Scheme 1a). ^177^Lu-SPN-GIP was designed to comprise radioactive ^177^Lu as a tracer for SPECT/CT imaging and also as a killer for tumor with a SPN to serve for PTT treatment of cancer. Such combination of different treatment techniques could complement each other, offering accurate monitoring and comprehensive therapeutic effects relative to PTT or RT alone. This is a simple yet effective approach to fabricate SPNs-based multifunctional theranostic nanoplatform for treatment of pancreatic tumor.

## Experimental section

### Preparation of ^177^Lu-SPN-GIP

^177^LuCl_3_-HCl solution (35 µL, 18.5–37MBq) was dissolved in 10 µL of 0.25 M sodium acetate (NaOAc). Then SPN-GIP solution (100 μg mL^−1^, 0.05 mL) was added into the above solution and then stirred at room temperature for 30 min. The crude product was centrifuged with 100 K ultrafiltration tube at 3000 r min^−1^ for 5 min three times. The radiochemical purity of ^177^Lu -SPN-GIP was measured by paper chromatography (mobile phase: pure water).

### PTT and RT of cells

CFPAC-1 cells were seeded in 96-well plates (5 × 10^3^ cells well^−1^) and cultured at 37 °C for 24 h. Then different concentrations of ^177^LuCl_3_ or ^177^Lu-SPN-GIP (0, 0.37, 3.70, 7.40, 11.10, 14.80 and 18.5 MBq mL^−1^) were added and incubated with cells for 24 h. After incubation, cells were washed and fresh medium was added to each well, and the cells were incubated for another 96 h. Cell viability was measured using the CCK-8 assay. For cell viability of different dose of ^177^Lu-SPN-GIP combined with PTT, after 24-h incubation of ^177^Lu-SPN-GIP (0, 0.37, 3.70, 7.40, 11.10, 14.80 and 18.5 MBq mL^−1^) mixing with 50 μg mL^−1^ SPN-GIP, cells were irradiated with 808 nm laser (1 W cm^−2^, 5 min), then incubation of fresh medium for another 24 h before measured using the CCK-8 assay. For live-dead cell imaging experiments, cells were firstly incubation with normal medium, 50 μg mL^−1^ of SPN-GIP, ^177^LuCl_3_ (1.11 MBq), and ^177^Lu-SPN-GIP (1.11 MBq) for 24 h and then with or without irradiated by an 808 nm laser at the power density of 1 W cm^−2^ for 5 min. Afterwards, cells were co-stained with a live/dead cell staining kit to monitor live and dead cells. The double staining kit contains acetoxymethyl ester of calcein to stain viable cells with green fluorescence, and propidium iodide to stain dead cells with red fluorescence.

### Therapeutic effect

Animal experiments were performed using 6 − 8 weeks old male BALB/c nude mice (Cavens, Changzhou, China). To create the pancreas cancer model, a single-cell suspension of 2 × 10^6^ CFPAC-1 cells in 75 μL of IMDM without serum was injected into the right leg of BALB/c nude mice. On the tenth day after subcutaneous inoculation, mice with tumor diameter at about 4 − 5 mm were selected for further studies. All animal experiments were conducted according to the animal research guidelines provided by the Animal Care and Use Committee at the Soochow University. Six groups of mice were received an intratumoral injection of SPNs (2 mg kg^−1^, 20 μL), SPNs (2 mg kg^−1^, 20 μL) with laser irradiation (808 nm, 1 W cm^−2^, 5 min), ^177^LuCl_3_ (1.11 MBq), ^177^Lu-SPN-GIP (1.11 MBq), ^177^Lu-SPN-GIP (1.11 MBq) with PTT and saline (n = 5), respectively. The excitation laser (808 nm) was generated from cnilaser YZ808KD1000-34F (1 W cm^−2^) and the temperature of tumor was controlled around 45 ℃ by the distance between the cnilaser and the tumor surface which was recorded by FLIR camera.

### SPECT/CT imaging

CFPAC-1 bearing mice (at t = 10-day after subcutaneous inoculation) were intratumorally injected with 1.11 ± 0.11 MBq of ^177^Lu-SPN-GIP, or ^177^LuCl_3_. SPECT/CT scans were conducted by a small animal SPECT/CT imaging system (U-SPECT/CT, MILabs, Netherlands) at different time points (0.5, 24, 48 and 96 h).

## Results and discussions

### Synthesis and characterization of SPN-GIP and ^177^Lu-SPN-GIP

**Fig. 1:**
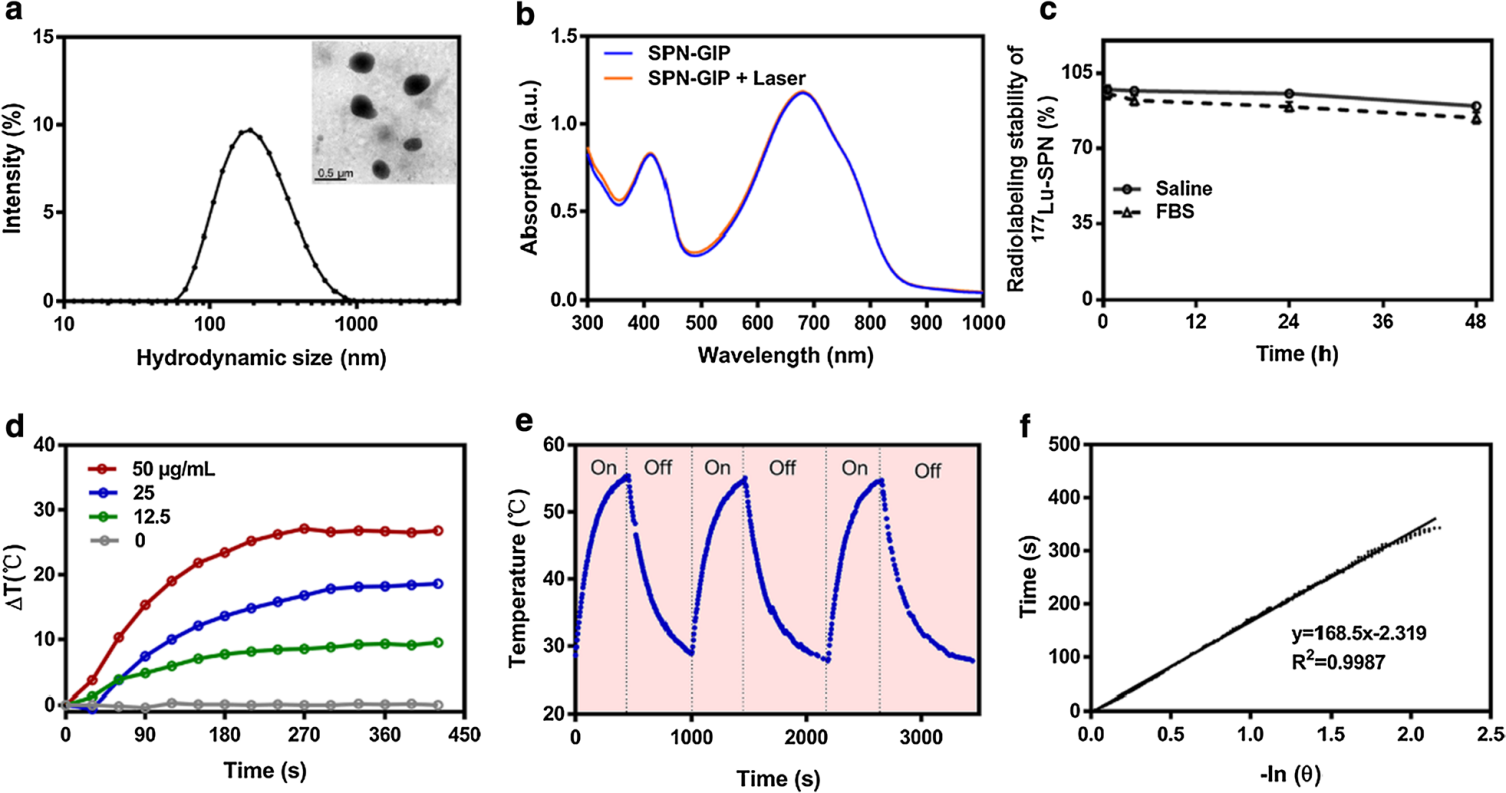
Synthesis and characterization. **a** Transmission electron microscopy (TEM) image of SPN-GIP and hydrodynamic size of SPN-GIP determined by DLS. **b** UV–vis spectra of SPN-GIP before and after light irradiation at 808 nm. **c** Stability of ^177^Lu-SPN-GIP in saline and FBS at various time points. **d** Temperature changes of SPN-GIP at different concentrations after 808 nm irradiation (1 W cm^−2^). **e** Three heating–cooling circles of SPN-GIP in the presence or absence of 808 nm irradiation. **f** Linear fitting curve of the cooling time of SPN-GIP as a function of negative logarithm of temperatures

Scheme 1a presents the synthetic procedures of SPN-GIP and ^177^Lu-SPN-GIP. SPN-GIP was prepared using a nanoprecipitation method. A semiconducting polymer poly[2,1,3-benzothiadiazole-4,7-diy1[4,4-bis(2-ethylhexyl)-4H-cyclopenta[2,1-b:3,4-b′] dithiophene-2,6-diyl]] (PCPDTBT) was encapsulated with functionalized amphiphilic polymer (GIP-PEG_12_-DSPE and DOTA-PEG_45_-DSPE) to obtain water-soluble SPN-GIP. Further radiolabeling of SPN-GIP was performed through chelating ^177^Lu with DOTA on its surface to get the final radioactive nanoagent ^177^Lu-SPN-GIP. Transmission electron microscopy (TEM) revealed the spherical morphology of SPN-GIP with an average size of 207.70 nm (Fig. 1a). The hydrodynamic diameter of SPN-GIP in PBS was measured to be 187.8 ± 2.26 nm (Fig. 1a). SPN-GIP had an absorption in the NIR region with a maximum peak at 680 nm, which was originated from PCPDTBT (Fig. 1b). SPN-GIP had no obvious change in absorption intensity after 808 nm laser irradiation, suggesting its excellent photostability for further PTT study (Fig. 1b). The radiolabeling efficiency of ^177^Lu-SPN-GIP was 86.36 ± 6.12% with a high radiochemical purity of 97.48 ± 1.90%. After a 48-h incubation, the radiochemical purity of ^177^Lu-SPN-GIP remained to be 89.78 ± 0.75% in saline and 84.22 ± 2.60% in FBS (Fig. 1c), indicating its suitable stability for in vivo study. The photothermal performance of SPN-GIP at various concentrations (0, 12.5, 25, and 50 μg mL^−1^) was investigated with an 808 nm laser at 0.5 W cm^−2^, which was recorded by an infrared thermal mapping apparatus (Additional file 1: Fig. S1). Under continuous laser irradiation at 808 nm, the temperature of SPN-GIP solutions gradually increased with increasing its concentrations. The maximum temperature increment of SPN-GIP at 12.5, 25, and 50 μg mL^−1^ after laser irradiation for 270 s reached about 8.5, 16.8, and 27.1 °C, respectively (Fig. 1d). In contrast, the temperature increment of PBS solution was only 0.3 °C with the same laser irradiation density and time, indicating the capability of SPN-GIP for PTT. Remarkably, the photothermal conversion efficiency (*η*) reached approximately 42.84%, [25, 38], which was comparable to the previously reported photothermal agents (Additional file 1: Table S1). After three cycles of heating–cooling manipulation, no reduction of maximum temperature at each cycle was observed, indicating the perfect photothermal stability of SPN-GIP (Fig. 1e, f).

### In vitro RT and PTT capability of SPN-GIP and ^177^Lu-SPN-GIP

**Fig. 2:**
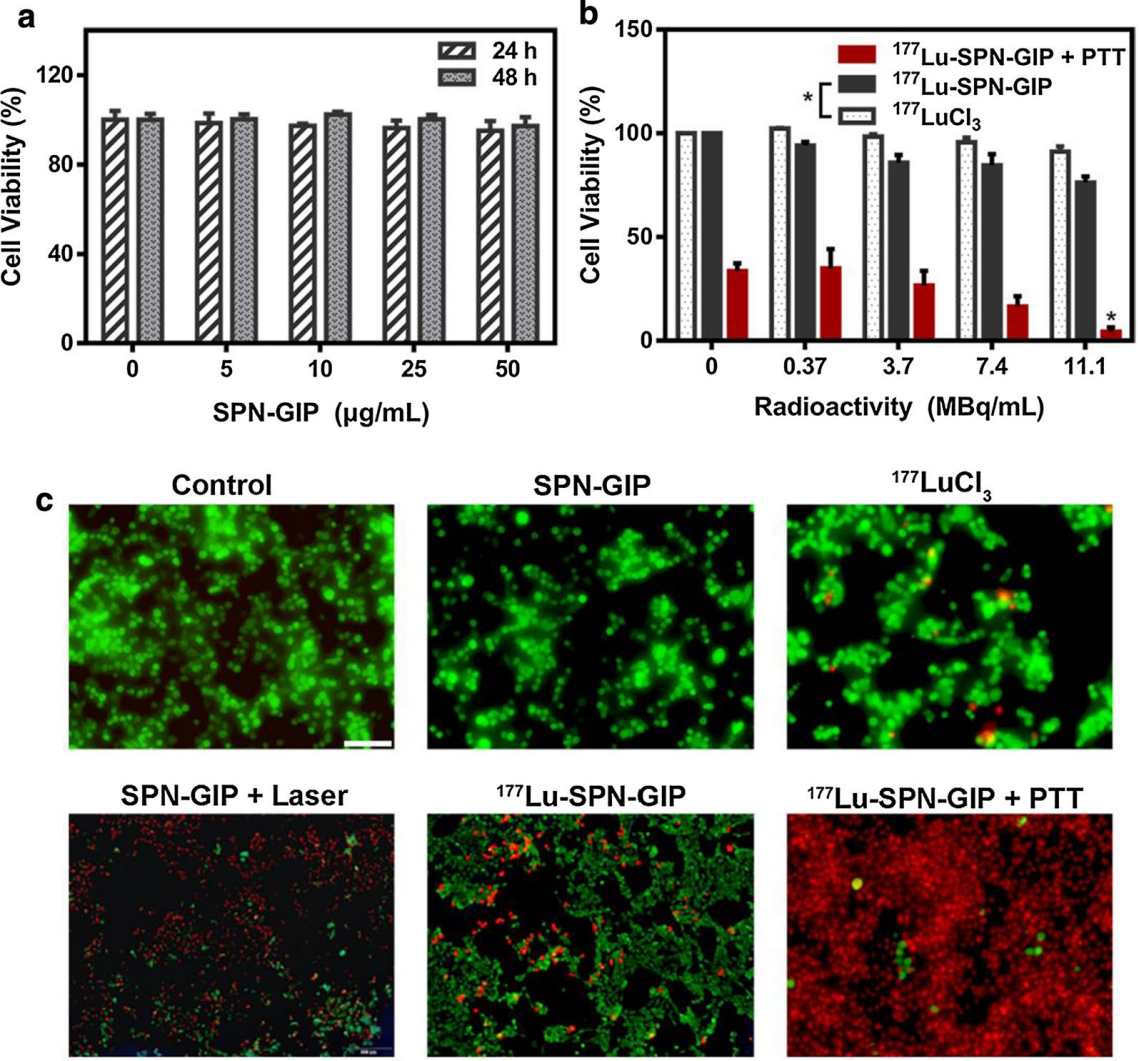
In vitro therapeutic study. **a** Cell viability of CFPAC-1 cells after incubation with SPN-GIP at various concentrations for 24 h and 48 h (the concentration of SPN-GIP represents the content of PCPDTBT). **b** The relative viabilities of CFPAC-1 cells after incubation with different radioactive doses of ^177^LuCl_3_, ^177^Lu-SPN-GIP for 24 h, or ^177^Lu-SPN-GIP along with PTT, followed by incubation with fresh medium for another 24 h. **c** Fluorescence imaging of CFPAC-1 cells after various treatment in (**b**) (the radioactive dosage is 1.11 MBq) and staining with Calcein AM/PI. The laser irradiation was conducted at a power density of 1 W cm^−2^ for 5 min. Scale bar: 200 µm

Before investigating the RT and PTT performance, cell biocompatibility of SPN-GIP towards CFPAC-1 cells was firstly tested using cell counting kit-8 (CCK8) assay. As shown in Fig. 2a, SPN-GIP exhibited no appreciable toxicity to cells even at a high concentration of 50 μg mL^−1^, showing its great biocompatibility. However, after labeling with ^177^Lu, ^177^Lu-SPN-GIP showed dose-dependent decrease in cell viability (Fig. 2b). In contrast, free ^177^LuCl_3_ showed a significant decrease in cancer cell killing ability, which probably because the nanoparticle is a better carrier in favor of the cell uptake of ^177^Lu and thereafter facilitates RT within cells (Fig. 2b, Additional file 1: Fig. S2a, b). Besides, if the incubation time was extended from 24 to 96 h after replacing the medium containing ^177^Lu-SPN-GIP with fresh medium, the cell viability could be apparently decreased, indicating RT necessitates enough time to exert the killing effect. Then, a combination of RT and PTT was further investigated. After co-incubation with SPN-GIP (50 μg mL^−1^) and ^177^Lu-SPN-GIP at different radioactive dosage ranging from 0 to 11.1 MBq mL^−1^ for 24 h, CFPAC-1 cells were irradiated by an 808 nm laser (1 W cm^−2^, 5 min) and then incubated for another 24 h. After convergence with PTT, cell viability could be inhibited more effectively relative to RT alone. Specifically, after treatment with PTT along with RT at the dosage of 11.1 MBq mL^−1^ (1.11 MBq, 0.1 mL), cell viability could be decreased to 4.33 ± 3.03%, which was 7.74-fold and 18.58-fold lower than those of PTT and RT alone, respectively (Fig. 2b). Such combination of RT and PTT was demonstrated to possess outstanding effect on destructing cancer cells, which was further validated by fluorescence imaging using calcine AM and propidium iodide (PI) to stain cells after different therapeutic treatments (Fig. 2c). Therefore, the combined RT and PTT offers remarkably enhanced killing effect on cancer cells relative to RT or PTT alone.

### In vivo therapeutic effect of combined RT and PTT

**Fig. 3:**
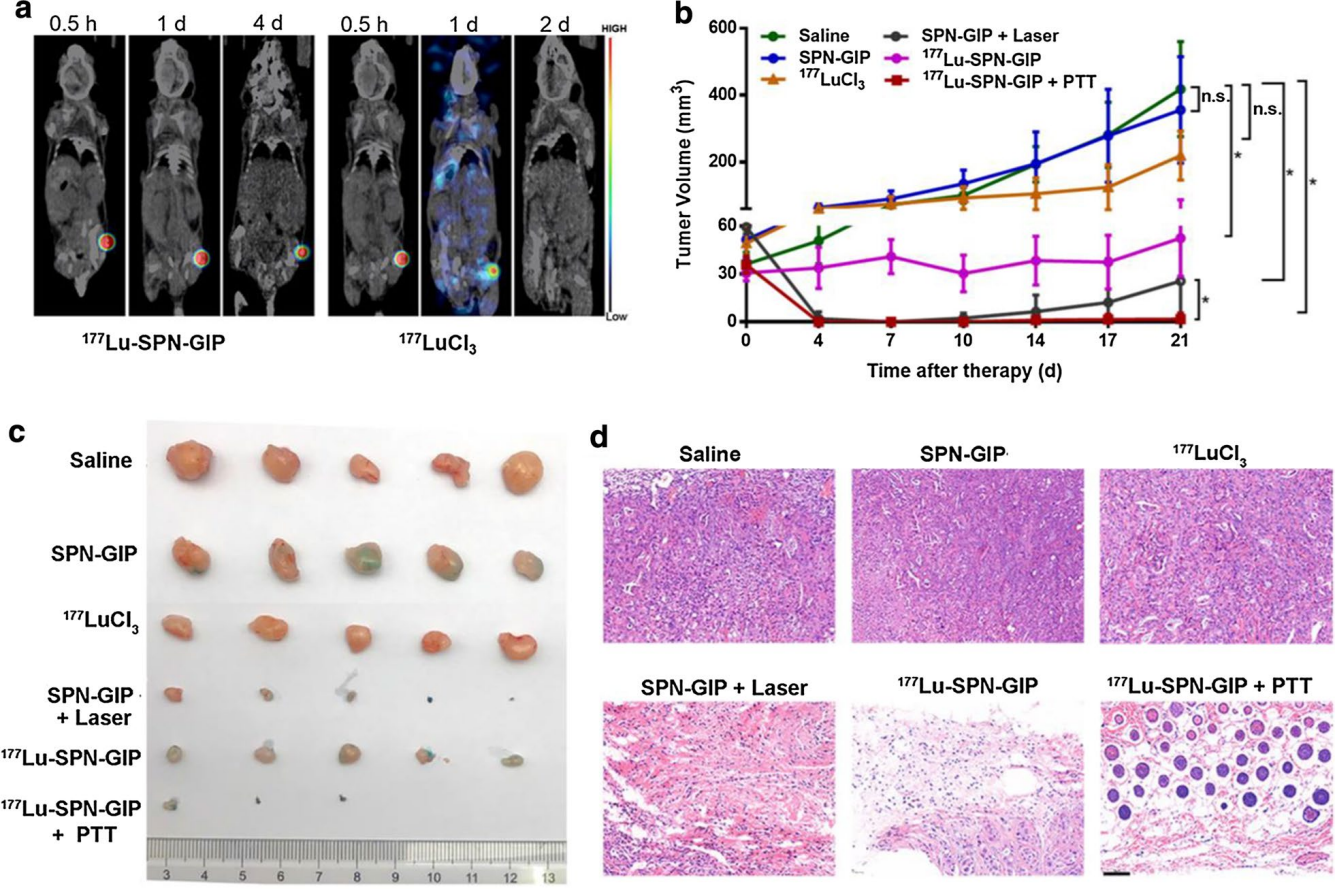
In vivo cancer therapeutic study. **a** SPECT/CT imaging of mice bearing CFPAC-1 tumors after intratumoral injection of ^177^Lu-SPN-GIP and free ^177^LuCl_3_ at the same radioactivity dose of 1.11 MBq. **b** The tumor growth curves of mice after indicated treatments. Six groups including saline treated mice (as control), SPN-GIP treated mice with or without laser irradiation (SPN-GIP group and SPN-GIP + Laser), ^177^LuCl_3_ treated mice (^177^LuCl_3_), and ^177^Lu-SPN-GIP treated mice with or without laser irradiation (^177^Lu-SPN-GIP and ^177^Lu-SPN-GIP + PTT) were used in this experiment (5 mice for each group). **c** Photos of tumors collected from different groups of mice at day 21 post-treatment. **d** H&E staining of tumor slices collected from different groups of mice at day 21 post treatment. Scale bar: 100 µm

As β-emitting radiopharmaceuticals for molecular RT, ^177^Lu is routinely used to treat patients suffering from tumors with somatostatin receptor overexpression in Europe, which invariably consists of 2 or 4 intravenous administrations of ^177^Lu-DOTATATE at a high dosage (7.4 GBq) [39, 40]. Such strategy can significantly improve progression-free survival, but it needs several injections due to the fast efflux-induced short retention of radiopharmaceuticals within tumor. In this study, after intratumoral injection of ^177^Lu-SPN-GIP, a strong radioactive signal was observed in tumor and the signal kept unchanged even at t = 4-day post-injection, indicating ^177^Lu-SPN-GIP had an excellent accumulation and retention effect in tumor region. By contrast, ^177^LuCl_3_-treated group showed a diffused radioactive signal in the whole body within 1-day post-injection and nearly all ^177^LuCl_3_ was excreted from the body at t = 2-day post-injection (Fig. 3a), further validating the SPNs as a perfect nanocarrier to favor the accumulation and retention of radiopharmaceuticals for therapy.

As it well known, heat causes irreversible damage to cancer cell membranes and initiates protein denaturation [16, 41]. In order to avoid unnecessary thermal effect to proximal normal tissues, the temperature of PTT was held around 45 °C [42, 43]. The temperature changes of the tumor area were recorded by IR thermal mapping apparatus. According to the IR thermographic images (Additional file 1: Fig. S3), the temperature at tumor area rapidly reached 45 °C after treatment with SPN-GIP under the laser irradiation for 30 s. The temperature of the tumor without treatment with SPNs presented subtle changes under the same irradiation conditions for 5 min, thereby demonstrating that the high in vivo photothermal effect of SPN-GIP.

Therapeutic efficacy of combined RT and PTT was then evaluated by monitoring the tumor growth after various treatments including saline, SPN-GIP, ^177^LuCl_3_, SPN-GIP with laser, ^177^Lu-SPN-GIP, and ^177^Lu-SPN-GIP with PTT (Fig. 3b–d). As shown in Fig. 3b, tumor volume in the saline-treated group grew over time. Though the tumor size of ^177^LuCl_3_-treated group at t = 7-day post-injection was slightly suppressed relative to those at t = 4-day post-injection probably due to the inherent killing effect of β-rays emitted by ^177^Lu, but there was no significant difference in comparison to the saline group post-treatment at day 21 (P = 0.2077). Compared with ^177^LuCl_3_-treated group, ^177^Lu-SPN-GIP showed obviously improved therapeutic capability in suppressing the tumor growth, attributing to increased tumor accumulation and retention effect of ^177^Lu-SPN-GIP relative to free ^177^LuCl_3_. Owing to ideal photothermal effect of SPN-GIP, SPN-GIP with PTT-treated group apparently inhibited the tumor growth after PTT while no therapeutic effect was observed for the group without laser irradiation (SPN-GIP group). Unfortunately, due to the low efficiency and incomplete killing of cancer cells, tumor recurrence was observed in both RT and PTT groups. By contrast, combined RT and PTT-treated group successfully inhibited the tumor growth within 21-day observation window. On the 21th day after treatment, mice of different groups were sacrificed and the residual tumor tissues were collected (Fig. 3c). Combine RT and PTT group presented the complete elimination of tumors while residual tumors were still observed in other groups receiving RT and PTT alone (Fig. 3c).

Tumors from various treatment groups were also collected for H&E staining (Fig. 3d). A mass of tumor cells was found almost in all the field of views in the control groups (including saline, SPN-GIP only, and ^177^LuCl_3_ group). As a comparison, the number of tumor cells was greatly reduced but a tiny of tumor cells was still observed in the RT and PTT alone-treated groups, indicating the incomplete killing effect of these groups. By contrast, no tumor cells were residual in combined PTT and RT group, which was in agreement with in vivo therapeutic result shown in Fig. 3b.

Besides, for mice in all groups, no significant weight loss was observed during 21-day observation period (Additional file 1: Fig. S4), and no noticeable histopathological abnormalities were found in heart, liver, spleen, lung, kidney, pancreas and intestine (Additional file 1: Fig. S5). In addition, serum biochemistry assay and blood glucose (GLU) test were also carried out for mice in all groups at t = 21-day post-treatment. There was no significant difference in GLU level between all treatment groups, suggesting there was no islet injury in all groups (Additional file 1: Fig. S5a). The liver (aspartate transaminase, AST and alanine transaminase, ALT) and kidney function markers (creatinine, CRE and blood urea nitrogen, URE) were measured and no significant difference was observed between treated groups and saline-treated group (Additional file 1: Fig. S5b, c). Therefore, these results verified the high therapeutic efficacy of synergistic PTT and RT with negligible risks towards the living organisms.

### Mechanistic study on therapeutic effect

**Fig. 4:**
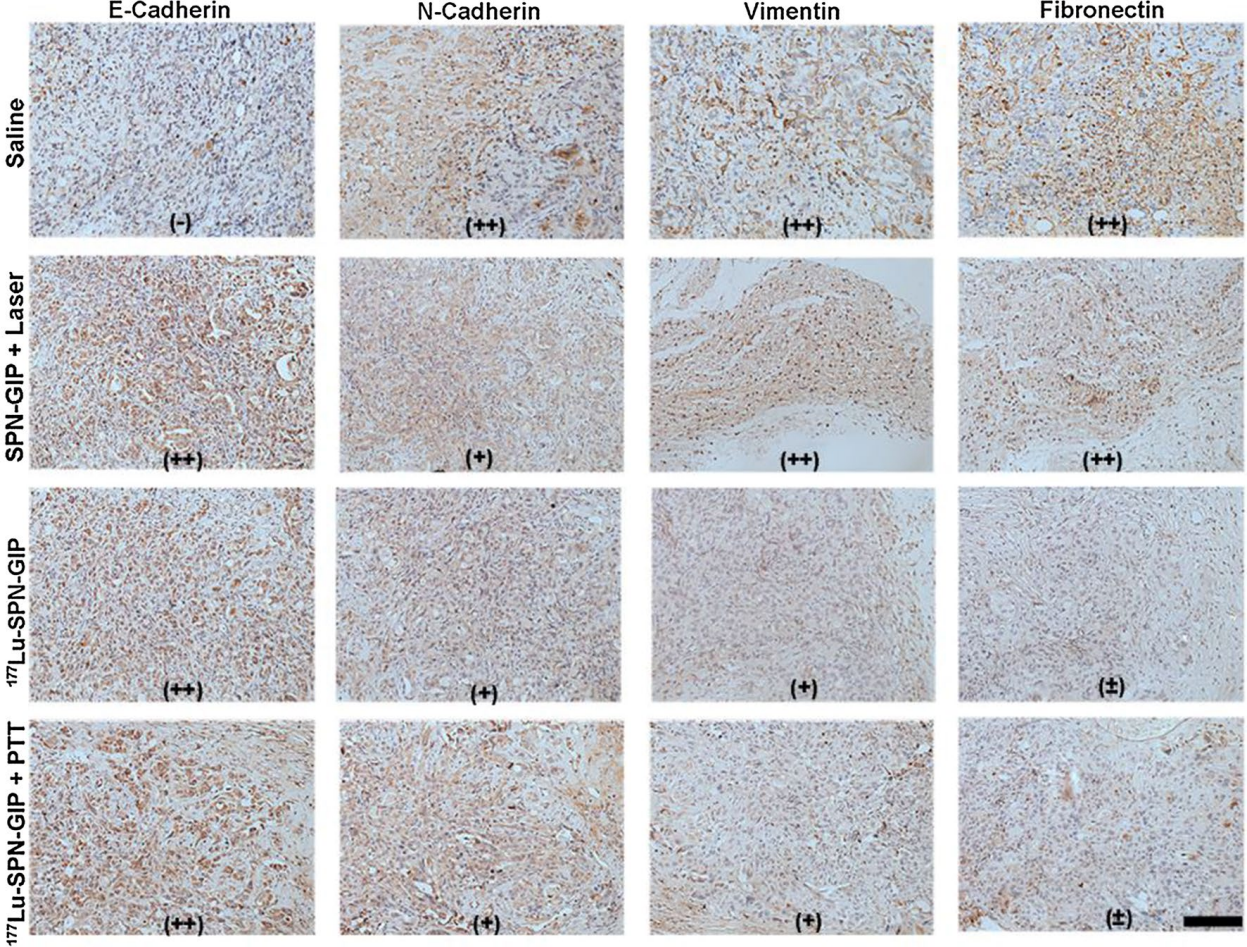
The study of EMT reversion after therapy. Immunohistochemistry of EMT markers (including E-Cadherin, N-Cadherin, Vimentin and Fibronectin) of tumor slices collected from different groups of mice at day 21 post treatment (including saline control, SPN-GIP with Laser, ^177^Lu-SPN-GIP, and ^177^Lu-SPN-GIP with PTT). +  + , + , ± ,-respectively represent strong positive, positive, weakly positive and negative expression of the marker. Scale bar: 200 μm

Epithelial-mesenchymal transition (EMT) is an important process to transform epithelial cells into mesenchymal cells, which is involved in tumorigenesis, invasion and metastasis [44, 45]. In the process of EMT, the epithelial marker E-cadherin is downregulated, while the mesenchymal markers N-cadherin, fibronectin, and vimentin are upregulated significantly [46]. By comparing with the expression of E-cadherin, N-cadherin, vimentin, and fibronectin in pancreatic tumor tissue with that in normal pancreas, EMT process was verified to occur in PDAC (Additional file 1: Fig. S6). Therefore, we utilized the method to evaluate the EMT process after various therapeutic treatments. Remarkably, the expression of the epithelial marker E-cadherin was upregulated meanwhile mesenchymal markers such as N-cadherin, vimentin, and fibronectin were downregulated in RT and combined RT/PTT-treated groups (Fig. 4). Such phenomenon verified that the EMT process was effectively reversed and thus resulted in reduced risk of tumor metastasis and invasion in both treatment groups [46]. Despite endowing the fast ablation of tumor, PTT-treated group showed similar expression of mesenchymal markers to saline-treated group, implying its inability to reverse EMT process. The imbalanced of upregulated epithelial E-cadherin in PTT-treated group was probably due to high temperature-induced the lysosome inactivation of the inhibition pathway or degradative pathway of E-cadherin [47]. Thus, the combination of PTT and RT not only facilitates efficient reversion of EMT process derived from RT but also enables fast tumor ablation contributed from PTT.

**Fig. 5:**
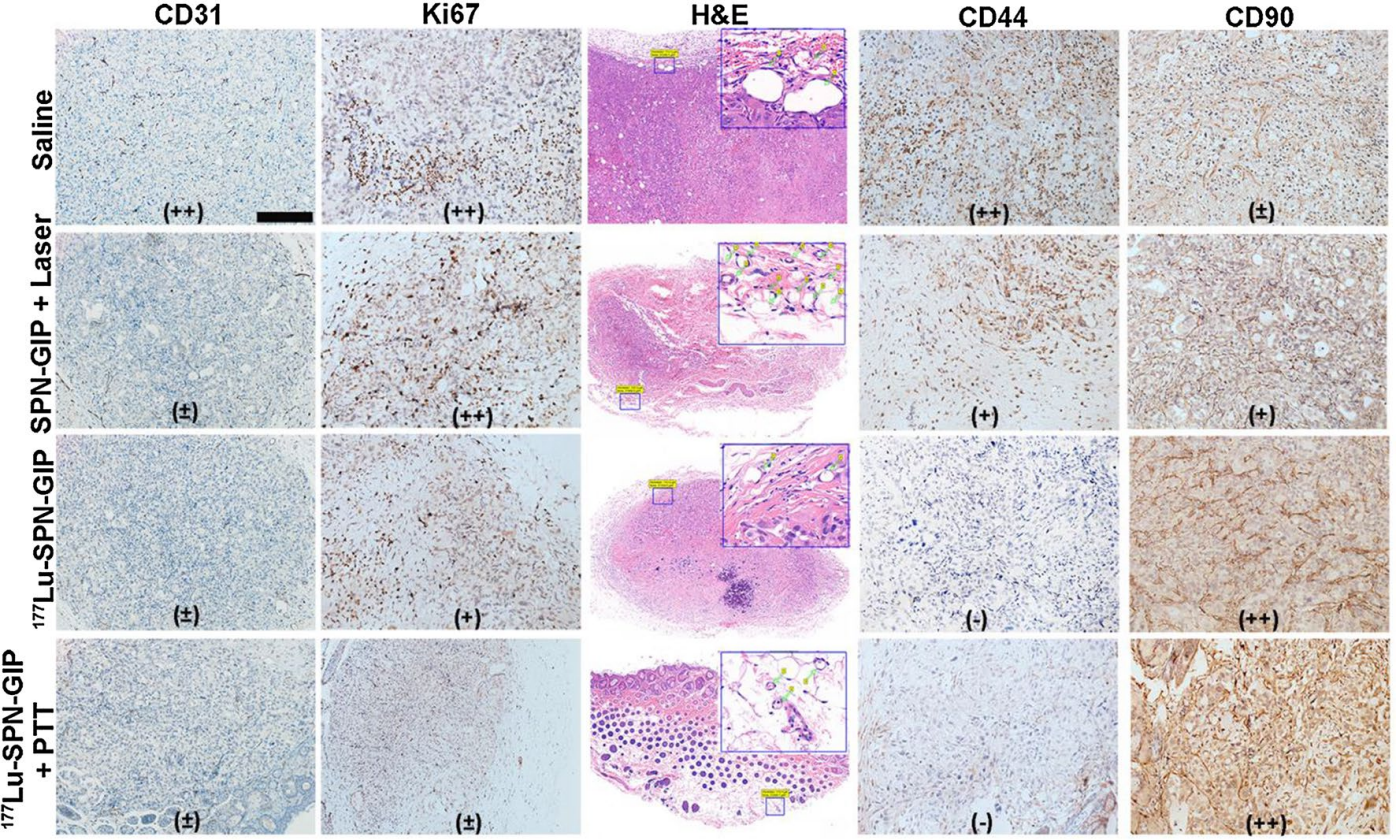
Histological evaluation after therapy. Histological evaluation of tumor after various treatment (CD31, Immunohistochemistry of Ki67, H&E staining, CD44, CD90). +  + , + , ± ,-respectively represent strong positive, positive, weakly positive and negative expression of the marker. Scale bar: 200 μm

Histological changes after different treatment were further analyzed (Fig. 5). As shown in Fig. 5, after treatment with PTT, RT, or the combination of PTT and RT, CD31 was changed from strong positive to weakly positive as compared with saline-treated group, indicating the successful anti-angiogenic effect of all therapeutic groups [48]. As a characteristic marker of tumor proliferation and aggressiveness, Ki67 was apparently decreased in the RT and RT/PTT-combined treatment group, indicating that the proliferation ability of tumor cells was greatly reduced [49]. However, there was no observable changes in the expression of Ki67 between PTT-treated group and saline-treated group although the tumors were rapidly ablated by elevated temperature. This was consistent with slow increase of tumor volume post-PPT treatment alone during observation window in Fig. 3. Such proliferating tendency post PTT treatment was possibly induced by its low efficiency to suppress the growth of the tumor margin and thus cause tumor proliferation and aggressiveness [14]. Thus, H&E staining was utilized to further investigate the tumor proliferation at its margin. As an expectation, an extensive generation of new capillaries was presented in the PTT-treated group relative to other groups. Such resulted abundance of capillaries could become a microenvironment conducive to tumor growth and spread. Besides, CD44 related to cancer stem cells (CSC) was also analyzed [46]. CSC is an important factor to predict the difficulty of tumor curability. Some studies showed that CSCs are closely related to EMT, resulted in expression of stem cell markers [45, 50, 51]. Compared with saline-treated group, the expression of CD44 was still observable in PTT-treated group, suggesting the existence of CSC and thereby implying a potential for recurrence. By contrast, the expression of CD44 decreased significantly in RT-treated and RT/PTT-combined-treated groups, validating the effective killing of CSC via ^177^Lu-induced damage of DNA double strands, which was also confirmed by positive immunofluorescence of γ-H_2_AX in cells and tissues of both groups (Additional file 1: Figure S7) [40]. In addition, CD90 expression was found increasing remarkably in the RT and RT/PTT-combined-treated groups, indicating the effective tumor-suppression while PTT-treated group showed its inferiority in the expression level [52]. All the data proved that combined PTT with RT not only achieved rapid tumor ablation derived from PTT, but also reversed EMT process and reduced the risk of invasiveness attributing to RT, providing a new attempt for effective tumor treatment.

## Conclusions and future perspectives

In summary, we developed the nano-radiopharmaceutical (^177^Lu-SPN-GIP) as a therapeutic agent for the combination of RT and PTT to treat pancreatic tumor. ^177^Lu-SPN-GIP was prepared by functionalizing SPNs with GIP and DOTA for targeting GIPR-overexpressing pancreatic tumors and chelating radionuclide ^177^Lu, respectively. The obtained ^177^Lu-SPN-GIP showed great stability in saline and FBS within 48 h. Comparing with free ^177^Lu, ^177^Lu-SPN-GIP exhibited significantly increased cellular uptake and thus improved in vitro RT efficacy. Taking advantages of the gamma-ray emitted from ^177^Lu, SPECT/CT imaging confirmed that ^177^Lu-SPN-GIP showed long-term retention in tumor. By virtue of the outstanding ability of tumor ablation of SPN and radiopharmaceutical function of ^177^Lu, in vivo combined PTT and RT based on ^177^Lu-SPN-GIP achieved a remarkable enhanced therapeutic effect of pancreatic tumor. Moreover, no noticeable toxicity or side effect was observed for ^177^Lu-SPN-GIP at the tested dose within 21 days. In this study, ^177^Lu-SPN-GIP was able to implement PTT and RT simultaneously to achieve synergistic therapy against cancer. Such strategy could improve therapeutic outcomes over traditional RT as it is able to ablate tumor with relatively lower doses of radiopharmaceuticals to reduce its side effects. Though remarkable therapeutic effects have been obtained, the underlying mechanisms and interactions of pathological environment respond to combined therapy need to be further explored, which is meaningful to guide the exploration and advances of more effective therapeutic strategies against tumors.

## Supplementary Information

**Additional file 1: Fig. S1.** IR thermal images of different concentration of SPN-GIP (0, 12.5, 25, and 50 µg mL^−1^) in the EP tube by 808 nm laser at different time point. **Fig. S2.** Cell culture. (a): Cells uptake of ^177^LuCl_3_ and ^177^Lu-SPN-GIP at different time points (1, 2, 4 and 8 h). (b): Cell viability at different dose of ^177^Lu-SPN-GIP after 24-h incubation then changed to fresh medium for another 24, 48, 72, and 96 h. (c): Confocal imagines of CFPAC-1 cells uptake SPN-GIP at different time points (pre as control, 1 2, 4, and 8 h). **Fig. S3.** IR thermal images of mice with intratumor injected at 3, 30, 60, 180, and 300 s under irradiation at the tumor region by 808nm laser at 1 W cm^−2^. **Fig. S4.** The body weight of mice in the six treatment groups. **Fig. S5.** The side effect analysis data of different groups of treatment mice. (a) The blood levels of GLU from treated and saline control mice (P = 0.6141). (b) AST and ALT levels in the blood (P = 0.2252 for AST, P = 0.3394 for ALT). (c) CRE and URE levels in the blood (P = 0.5635 for CRE, P = 0.2176 for URE). (d) Hematoxylin and eosin (H&E)-stained slices of heart, liver, spleen, lung, kidney, pancreas, and intestines tissues of mice after PTT, RT, and saline treatments (at day-21 after intratumor). Scale bar: 50 μm. **Fig. S6.** Immunohistochemistry of EMT markers expression in normal pancreas and pancreatic cancer. Scale bar: 50 μm. **Fig. S7.** (a) Confocal imaging of CFPAC-1 cells after treatment with ^177^Lu-SPN-GIP with or without laser followed by staining with γH_2_AX. (b) Immunofluorescence imaging of tumor sections (10 μm) after staining with γH_2_AX.  **Table S1.** Summary of optical contrast agents for PTT agents (ΔT = temperature increase; η = photothermal conversion efficiency).

## Data Availability

All data related to the manuscript are available in the manuscript and supporting information.
