# Supplementary material for: Semiconducting polymer nano-radiopharmaceutical for combined radio-photothermal therapy of pancreatic tumor
Source: J Nanobiotechnology. 2021 Oct 24;19:337. doi: 10.1186/s12951-021-01083-0 (PMC8543882; doi:10.1186/s12951-021-01083-0)
Supplement: Supplementary file 1 — Additional file 1: Fig. S1. IR thermal images of different concentration of SPN-GIP (0, 12.5, 25, and 50 µg mL−1) in the EP tube by 808 nm laser at different time point. Fig. S2. Cell culture. (a): Cells uptake of 177LuCl3 and 177Lu-SPN-GIP at different time points (1, 2, 4 and 8 h). (b): Cell viability at different dose of 177Lu-SPN-GIP after 24-h incubation then changed to fresh medium for another 24, 48, 72, and 96 h. (c): Confocal imagines of CFPAC-1 cells uptake SPN-GIP at different time points (pre as control, 1 2, 4, and 8 h). Fig. S3. IR thermal images of mice with intratumor injected at 3, 30, 60, 180, and 300 s under irradiation at the tumor region by 808nm laser at 1 W cm−2. Fig. S4. The body weight of mice in the six treatment groups. Fig. S5. The side effect analysis data of different groups of treatment mice. (a) The blood levels of GLU from treated and saline control mice (P = 0.6141). (b) AST and ALT levels in the blood (P = 0.2252 for AST, P = 0.3394 for ALT). (c) CRE and URE levels in the blood (P = 0.5635 for CRE, P = 0.2176 for URE). (d) Hematoxylin and eosin (H&E)-stained slices of heart, liver, spleen, lung, kidney, pancreas, and intestines tissues of mice after PTT, RT, and saline treatments (at day-21 after intratumor). Scale bar: 50 μm. Fig. S6. Immunohistochemistry of EMT markers expression in normal pancreas and pancreatic cancer. Scale bar: 50 μm. Fig. S7. (a) Confocal imaging of CFPAC-1 cells after treatment with 177Lu-SPN-GIP with or without laser followed by staining with γH2AX. (b) Immunofluorescence imaging of tumor sections (10 μm) after staining with γH2AX. Table S1. Summary of optical contrast agents for PTT agents (ΔT = temperature increase; η = photothermal conversion efficiency). [file 12951_2021_1083_MOESM1_ESM.docx]

Supporting information

Semiconducting Polymer Nano-Radiopharmaceuticals for Combined Radio-Photothermal Therapy of Pancreatic Tumor

Xiumin Shi ^a,b^, Qing Li ^a^ , Chuan Zhang ^a^, Hailong Pei ^a^, Guanglin Wang ^a^, Hui Zhou ^a^, Longfei Fan ^a^, Kai Yang^a^, Bo Jiang ^c,*^, Feng Wang ^b,*^, Ran Zhu ^a,*^

^a^ State Key Laboratory of Radiation Medicine and Protection, School of Radiation Medicine and Protection & School for Radiological and Interdisciplinary Sciences (RAD-X), Collaborative Innovation Center of Radiation Medicine of Jiangsu Higher Education Institutions, Soochow University, Suzhou, Jiangsu 215123, China.

^b^ Department of Nuclear Medicine, Nanjing First Hospital, Nanjing Medical University, Nanjing 210006, China.

^c^ Department of Neuro-oncology, Cancer Center, Beijing Tiantan Hospital, Capital Medical university, Beijing 100071, China.

E-mail: jiangboprof@163.com; fengwangcn@hotmail.com; zhuran@suda.edu.cn.

**Materials and Methods**

**General.** Radionuclide lutetium-177 (^177^LuCl_3_) was purchased from Curium Pharma and ITG Isotopes Technologies Garching GmbH. Glucose-dependent insulinotropic polypeptide (GIP, H-Cys-Tyr-(D-Ala)-Glu-Gly-Thr-Phe-Ile-Ser-Asp-Tyr-Ser-Ile-Ala-Met-Asp-Lys-Ile-His-Gln-Gln-Asp-Phe-Val-Asn-Trp-Leu-Leu-Ala-Gln-Lys-OH) was purchased from GL Biochem Company Limited (GLS, Shanghai, China). Poly [2,6-(4,4-bis-(2-ethylhexyl)‐4H-cyclopenta[2,1-b;3,4-b′]dithiophene)-alt-4,7(2,1,3-benzothiadiazole)] (PCPDTBT) was purchased from Sigma (USA). Maleimide (polyethyleneglycol) distearoylphosphatidylethanolamine (MAL-PEG_12_-DSPE) and 1,4,7,10-Tetraazacyclododecane-1,4,7,10-tetraacetic acid mono-N-hydroxysuccinimide ester (DOTA-NHS ester) were purchased from Macrocyclics (USA). 1,2-distearoyl-sn-glycero-3-phosphoethanolamine-N-[amino(polyethylene glycol)] (NH_2_-PEG_45_-DSPE) and other chemicals were obtained from chemical industries (China). In the process of preparing DOTA-SPN-GIP, functionalized amphiphilic polymer DSPE-PEG includes 66.7_w/w_% DSPE-PEG_12_-Mal and 33.3_w/w_% DSPE-PEG_45_-NH_2._ A CRC-25R Curiemeter dose calibrator (CAPINTEC.INC, USA) was used for radioactivity measurements.

**Synthesis of semiconducting polymer nanoparticles with glucose-dependent insulinotropic polypeptide (SPN-GIP).**

SPN-GIP was synthesized as follows: Firstly, glucose-dependent insulinotropic polypeptide (GIP) (1 mg mL^-1^ in PBS) and MAL-PEG_12_-DSPE (0.2 mg mL^-1^ in PBS) were mixed with TCEP (1.41 mg) at pH 7.4, and then stirred overnight at room temperature. Afterwards, the mixture was dialyzed to remove salt and small molecular impurities. Pure GIP-PEG_12_-DSPE was obtained after lyophilization and stored at -20 ^o^C. Secondly, NH_2_-PEG_45_-DSPE (260 µg dissolved in 26 µL DMSO) was mixed with 44 µg of DOTA-NHS and then stirred for 2 h. DOTA-PEG_45_-DSPE was obtained through dialysis and lyophilization. Finally, SPN-GIP nanocomplex was prepared via nanoprecipitation. A mixture of GIP-PEG_12_-DSPE (600 µg), DOTA-PEG_45_-DSPE (300 µg) and PCPDTBT (50 µg) dissolved in THF (200 µL) was rapidly injected into water (1.8 mL) under ultrasonication. The mixed solution was under continuous sonication for 15 min. Afterwards, the obtained blue clarity solution was degassed by nitrogen atmosphere to remove THF. The obtained SPN-GIP solution was centrifuged twice with 100 K ultrafiltration tube at 3000 r min^-1^ for 5 min, concentrated to a volume of 200 µL and stored at 4 ^o^C.

**Preparation of ^177^Lu-SPN-GIP.** ^177^LuCl_3_-HCl solution (35 µL, 18.5 MBq ~ 37 MBq) was dissolved in 10 µL of 0.25 M sodium acetate (NaOAc). Then SPN-GIP solution (100 μg mL^-1^, 0.05 mL) was added into the above solution and then stirred at room temperature for 30 min. The crude product was centrifuged with 100 K ultrafiltration tube at 3000 r min^-1^ for 5 min three times. The radiochemical purity of ^177^Lu -SPN-GIP was measured by paper chromatography (mobile phase: pure water).

**Cell culture and in vivo model**

Human cystic fibrosis pancreatic adenocarcinoma cells (CFPAC-1) were obtained from the American Type Culture Collection (ATCC, China) and cultured under the guidance of ATCC. Animal experiments were performed using 6 - 8 weeks old male BALB/c nude mice (Cavens, Changzhou, China). 2 × 10^6^ CFPAC-1 cells were injected into the right leg of BALB/c nude mice for building the pancreas models. After subcutaneous inoculation for 10 days, mice with tumor diameter from approximately 4 mm to 5 mm were selected for next experiments. All animal experiments were conducted according to the animal research guidelines provided by the Animal Care and Use Committee at the Soochow University. The animals were raised in the recommendations of the National Institute of Health and Soochow University institutional guidelines.

**Photothermal effect measurements.**

A volume of SPNs aqueous solution (50 μg mL^-1^, 0.2 mL) was introduced into eppendorf (EP) tube, which was irradiated with the laser (808 nm) for 10 min at a power density of 1 W cm^-2^. Pure water was used as a negative control. A thermal imaging temperature sensor (FLIR, A65, American) was used to measure the temperature. The infrared imager was closed to SPN aqueous solution, and the temperature pattern could be recorded at 1 s intervals by the digital thermometer. The photothermal conversion efficacy (*η*) of SPN-GIP was calculated according to previously reported methods as follows:

$\eta=\frac{hA\Delta T_{max}-Q_{s}}{I\left( 1-{10}^{-A808} \right)}$ (1)

where h is the coefficient of heat transfer, A is the container surface area, ΔT_max_ is the maximum temperature change of SPN solution, I is the power density of the NIR laser, A_808_ is the absorbance of the solution of SPN at 808 nm, and Qs is the heat associated with the light absorbance of the water.

**Cytotoxicity of SPNs.**

Cell Counting Kit-8 (CCK-8) (Dojingdo, Japan) assays were used for evaluation of in vitro cell viability. Human cystic fibrosis pancreatic adenocarcinoma (CFPAC-1) cells were cultured in Iscove’s Modified Dulbecco’s Medium (IMDM) with 10% fetal bovine serum and 1% penicillin/streptomycin. The cells were seeded into 96-well cell culture plates. The density of cells was 1 × 10^4^ well^-1^. Then, the media were removed and replaced with SPNs at various concentrations (0, 5, 10, 25, and 50 μg mL^-1^) for 24 h or 48 h’s incubation at 37 °C. After incubation, 10 μL of CCK-8 solution was added into the cells and incubated for another 1 h. Absorbance (OD_450 nm_) of each well was measured by microplate reader and the cell viability was calculated via the following equation (At is the mean absorbance value of the treatment group, Ac is the mean absorbance value of the control group and A_0_ is the mean absorbance value of blank control which includes medium and 10 μL of CCK-8 solution):

Cell viability % = $\frac{At-A0}{Ac-A0}\times100\%$.

**PTT and RT of cells.** CFPAC-1 cells were seeded in 96-well plates (5 × 10^3^ cells well^-1^) and cultured at 37 °C for 24 h. Then different concentrations of ^177^LuCl_3_ or ^177^Lu-SPN-GIP (0, 0.37, 3.70, 7.40, 11.10, 14.80 and 18.5 MBq mL^-1^) were added and incubated with cells for 24 h. After incubation, cells were washed and fresh medium was added to each well, and the cells were incubated for another 96 h. Cell viability was measured using the CCK-8 assay. For cell viability of different dose of ^177^Lu-SPN-GIP combined with PTT, after 24-h incubation of ^177^Lu-SPN-GIP (0, 0.37, 3.70, 7.40, 11.10, 14.80 and 18.5 MBq mL^-1^) mixing with 50 μg mL^-1^ SPN-GIP, cells were irradiated with 808 nm laser (1 W cm^-2^, 5 min), then incubation of fresh medium for another 24 h before measured using the CCK-8 assay. For live-dead cell imaging experiments, cells were firstly incubation with normal medium, 50 μg mL^-1^ of SPN-GIP, ^177^LuCl_3_ (1.11 MBq), and ^177^Lu-SPN-GIP (1.11 MBq) for 24 h and then with or without irradiated by an 808 nm laser at the power density of 1 W cm^-2^ for 5 min. Afterwards, cells were co-stained with a live/dead cell staining kit to monitor live and dead cells. The double staining kit contains acetoxymethyl ester of calcein to stain viable cells with green fluorescence, and propidium iodide to stain dead cells with red fluorescence.

**Therapeutic effect.** Animal experiments were performed using 6−8 weeks old male BALB/c nude mice (Cavens, Changzhou, China). To create the pancreas cancer model, a single-cell suspension of 2 × 10^6^ CFPAC-1 cells in 75 μL of IMDM without serum was injected into the right leg of BALB/c nude mice. On the tenth day after subcutaneous inoculation, mice with tumor diameter at about 4−5 mm were selected for further studies. All animal experiments were conducted according to the animal research guidelines provided by the Animal Care and Use Committee at the Soochow University. Six groups of mice were received an intratumoral injection of SPNs (2 mg kg^-1^, 20 μL), SPNs (2 mg kg^-1^, 20 μL) with laser irradiation (808 nm, 1 W cm^-2^, 5 min), ^177^LuCl_3_ (0.11 MBq), ^177^Lu-SPN-GIP (0.11 MBq), ^177^Lu-SPN-GIP (0.11 MBq) with PTT and saline (n = 5), respectively. The excitation laser (808 nm) was generated from cnilaser YZ808KD1000-34F (1 W cm^-2^) and the temperature of tumor was controlled around 45 ℃ by the distance between the cnilaser and the tumor surface which was recorded by FLIR camera.

**SPECT/CT imaging.** CFPAC-1 bearing mice (at t = 10-day after subcutaneous inoculation) were intratumorally injected with 1.11 ± 0.11 MBq of ^177^Lu-SPN-GIP, or ^177^LuCl_3_. SPECT/CT scans were conducted by a small animal SPECT/CT imaging system (U-SPECT/CT-Ⅱ, MILabs, Netherlands) at different time points (0.5, 24, 48 and 96 h).

**Histopathological evaluation and immunohistochemistry.**

For histological analysis, the major organs (including tumor, heart, liver, spleen, lung, kidney, pancreas, and intestine) were fixed in 10% formalin, then embedded in paraffin. Slices of organs from the mice were stained with hematoxylin and eosin (H&E). The histological sections were imaged by an optical microscope (Olympus, Japan). For immunohistochemistry (IHC), sections were incubated with EDTA (pH = 9.0) to repair and unmask antigens, then incubated with primary antibodies (Ki67, N-Cad, E-Cad, Vimentin, Fibronectin, CD31, CD44, and CD90) and DAB kit overnight at 4 °C, followed by incubation with secondary antibodies for 1 h at room temperature.


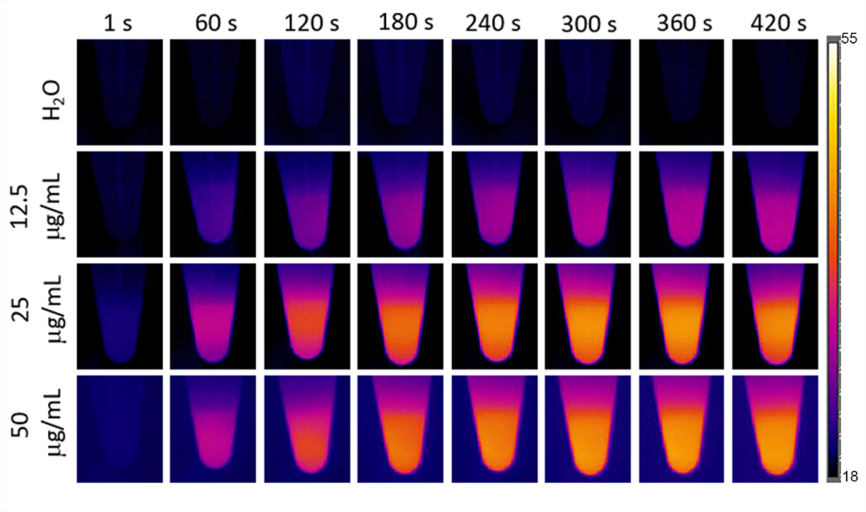


**Fig. S1**. IR thermal images of different concentration of SPN-GIP (0, 12.5, 25, and 50 µg mL^-1^) in the EP tube by 808nm laser at different time point.


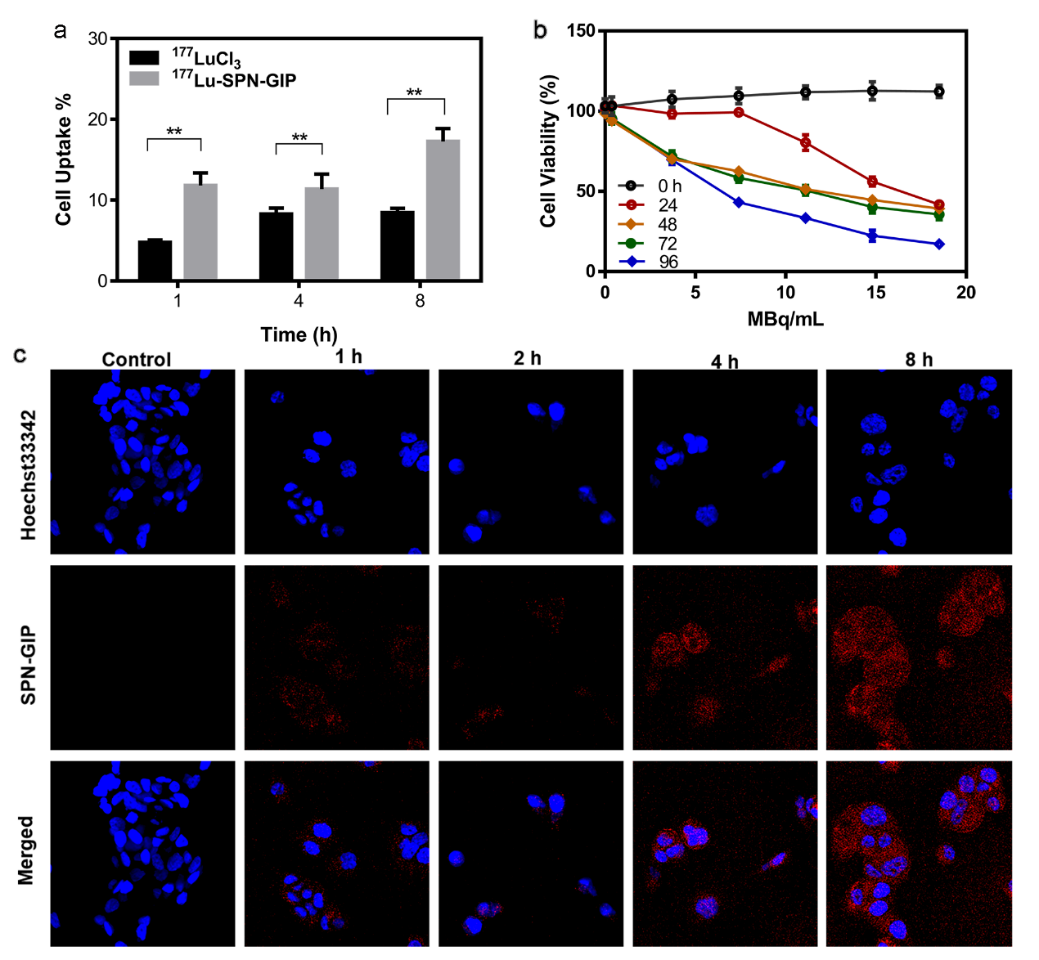


**Fig. S2**. Cell culture. (a): Cells uptake of ^177^LuCl_3_ and ^177^Lu-SPN-GIP at different time points (1, 2, 4 and 8 h). (b): Cell viability at different dose of ^177^Lu-SPN-GIP after 24-h incubation then changed to fresh medium for another 24, 48, 72, and 96 h. (c): Confocal imagines of CFPAC-1 cells uptake SPN-GIP at different time points (pre as control, 1 2, 4, and 8 h).


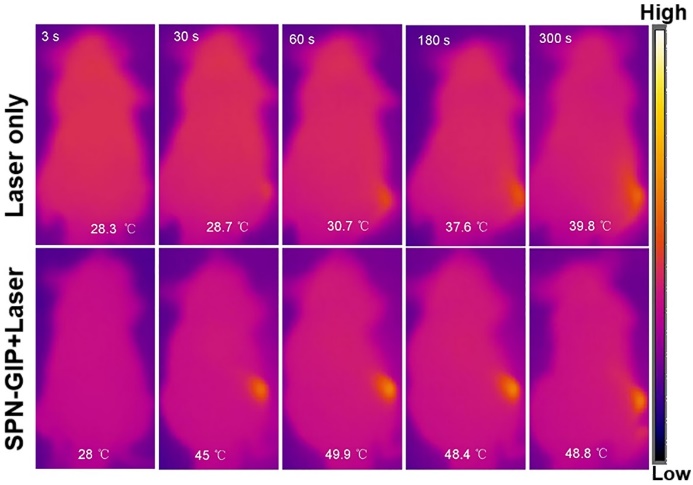


**Fig. S3**. IR thermal images of mice with intratumor injected at 3, 30, 60, 180, and 300 s under irradiation at the tumor region by 808nm laser at 1 W cm^−2^.


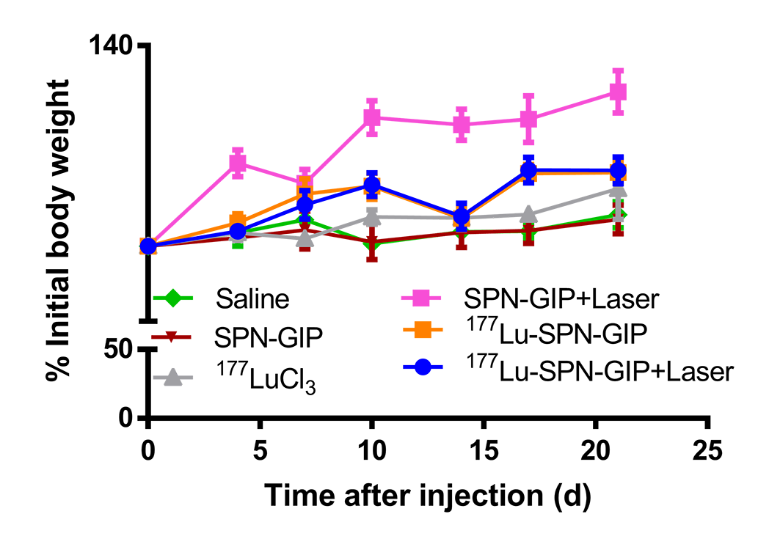


**Fig. S4.** The body weight of mice in the six treatment groups.


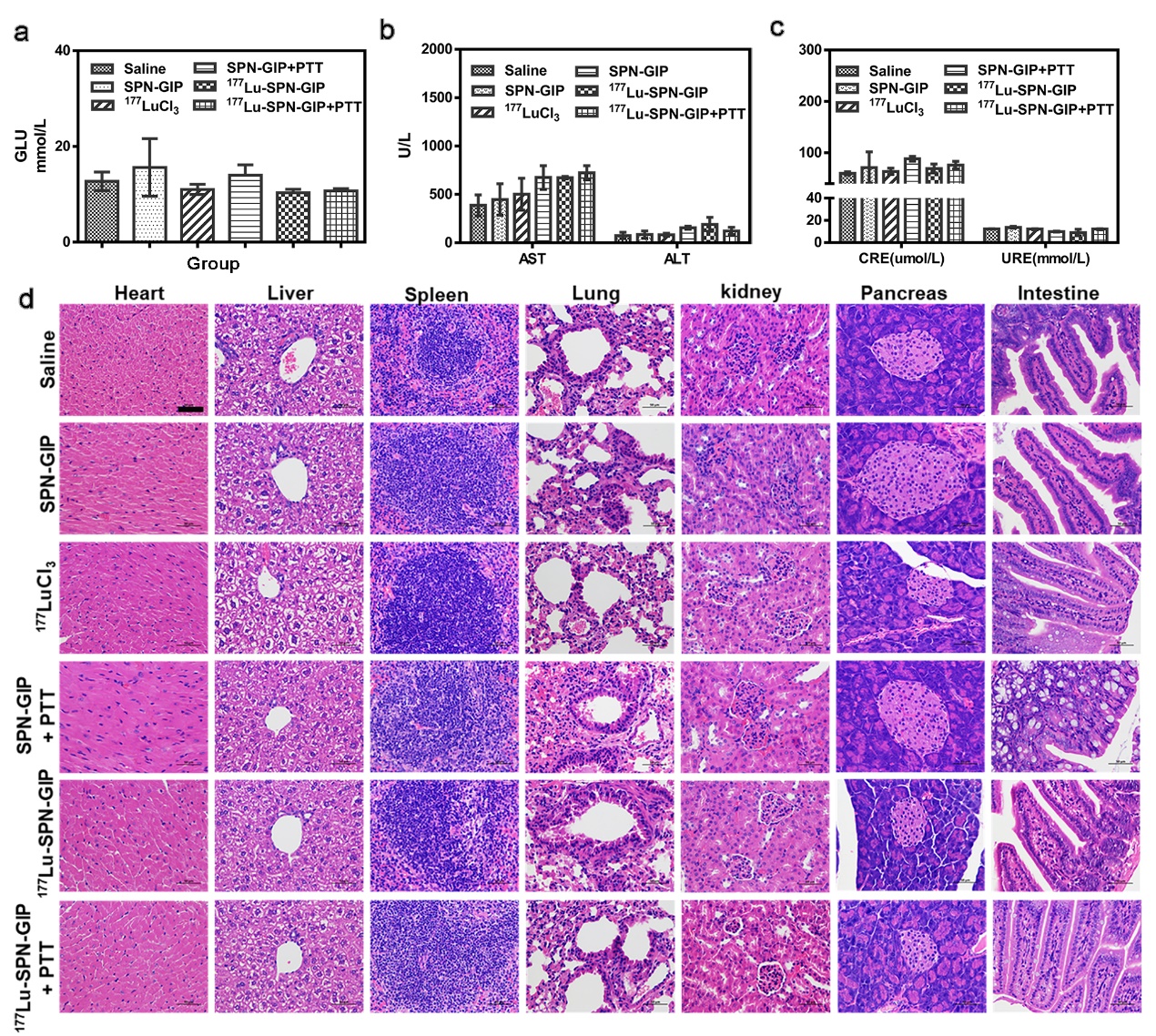


**Fig. S5**. The side effect analysis data of different groups of treatment mice. (a) The blood levels of GLU from treated and saline control mice (P = 0.6141). (b) AST and ALT levels in the blood (P = 0.2252 for AST, P = 0.3394 for ALT). (c) CRE and URE levels in the blood (P = 0.5635 for CRE, P = 0.2176 for URE). (d) Hematoxylin and eosin (H&E)-stained slices of heart, liver, spleen, lung, kidney, pancreas, and intestines tissues of mice after PTT, RT, and saline treatments (at day-21 after intratumor). Scale bar: 50 μm.


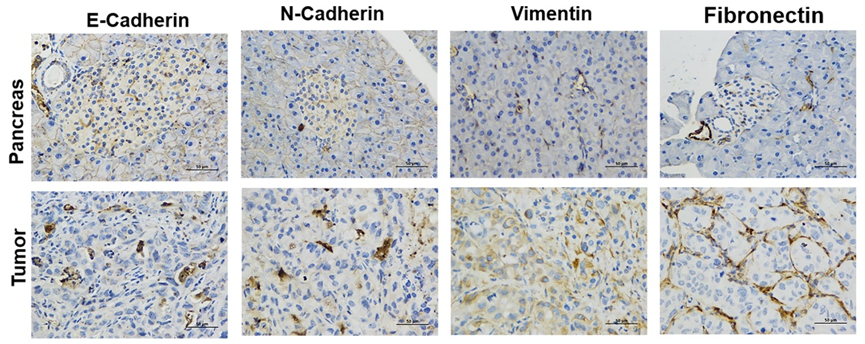


**Fig. S6**. Immunohistochemistry of EMT markers expression in normal pancreas and pancreatic cancer. Scale bar: 50 μm.


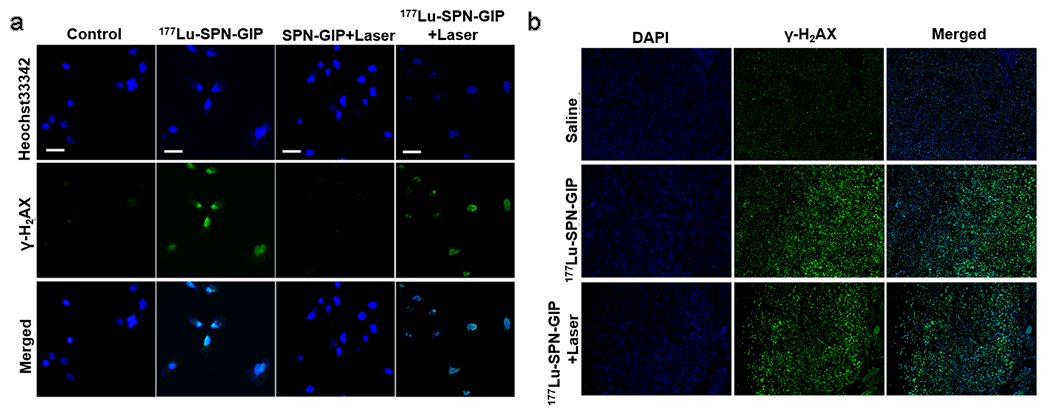


**Fig. S7**. (a) Confocal imaging of CFPAC-1 cells after treatment with ^177^Lu-SPN-GIP with or without laser followed by staining with γH_2_AX. (b) Immunofluorescence imaging of tumor sections (10 μm) after staining with γH_2_AX.

**Table S1**. Summary of optical contrast agents for PTT agents (ΔT = temperature increase; *η* = photothermal conversion efficiency).

| Agents | Laser irradiation | PTT performance | Ref. |
| --- | --- | --- | --- |
| Rattle-like Fe_3_O_4_@CuS–PEG | 1064 nm (3 W cm^−2^, 15 min) | ΔT = ≈24 °C (300 ppm Cu), *η* = 19.2% | ^1^ |
| Rattle-like Fe_3_O_4_@CuS–PEG | 808 nm (6 W cm^−2^, 15 min) | ΔT = ≈24 °C (300 ppm Cu), *η* = 15.7% | [1] |
| Rattle-like Fe_3_O_4_@Au/Ag | 1064 nm (3 W cm^−2^, 15 min) | ΔT = 34.7 °C (200 ppm Au), *η* = 28.28% | [2] |
| Cu_2‑x_Se | 800 nm (2 W cm^−2^, 5 min) | ΔT = 22 °C, *η* = 22% | [3] |
| Cu_9_S_5_ | 980 nm (0.51 W cm^−2^, 10 min) | ΔT = 15.1 °C (40 ppm f Cu_9_S_5_ NCs), *η* = 25.7% | [4] |
| PEGylated Cu_3_BiS_3_ | 1064 nm (1 W cm^−2^, 10 min) | ΔT = 27 °C (150 ppm of Bi), *η* = 40.7% | [5] |
| liposomal indocyanine green (ICG) composition | 808 nm (1.1 W cm^−2^, 1 min ) | ΔT = 15 °C, *η* = 8.99% | [6] |
| RC-bovine serum albumin (BSA) complement | 915 nm, (1.0 W cm^−2^, 10 min) | ΔT = 26 °C, *η* = 28.7% | [7] |
| DPP-TPA NPs | 660 nm, (1 W cm^−2^, 10 min) | ΔT = 10 °C (80 μg/mL), *η* = 34.5% | [8] |
| TPP-G-FF | 635 nm, (1.2 W cm^−2^,10 min) | ΔT = 35 °C, *η* = 54.2% | [9] |
| PPy@Fe_2_O_3_ | 808 nm, (0.25 W cm^−2^, 5 min) | ΔT = 33.5 °C, *η* = 39.2% | [10] |

**REFERENCES**

[1] Z.-C. Wu, W.-P. Li, C.-H. Luo, C.-H. Su, C.-S. Yeh, *Adv.* Funct. Mater. 25 (2015) 6527–6537.

[2] M.F. Tsai, C. Hsu, C.S. Yeh, et al., ACS Appl. Mater. Interfaces. 10 (2018) 1508-1519.

[3] C.M. Hessel, V. Pattani, M. Rasch, et al, Nano Lett. 11 (2011) 2560-2566.

[4] Q. Tian, F. Jiang, R. Zou, et al., ACS Nano*.* 5 (2011) 9761-9771.

[5] A. Li, X. Li, X. Yu, et al., Biomaterials*.* 112 (2017), 164-175.

[6] H.J. Yoon, H.S. Lee, J.Y. Lim, J.H. Park, ACS Appl. Mater. Interfaces. 9 (2017) 5683-5691.

[7] B. Zhou, Y. Li, G. Niu, et al., ACS Appl. Mater. Interfaces. 8 (2016) 29899-29905.

[8] Y. Cai, P. Liang, Q. Tang, et al., ACS Nano. 11 (2017) 1054-1063.

[9] Q. Zou, M. Abbas, L. Zhao, et al., J. Am. Chem. Soc. 139 (2017) 1921-1927.

[10] Q. Tian, Q. Wang, K.X. Yao, et al., Small*.* 10 (2014) 1063-1068.
